# Ultrasound Microbubble–Facilitated Inner Ear Delivery of Gold Nanoparticles Involves Transient Disruption of the Tight Junction Barrier in the Round Window Membrane

**DOI:** 10.3389/fphar.2021.689032

**Published:** 2021-06-28

**Authors:** Yi-Chun Lin, Cheng-Ping Shih, Hsin-Chien Chen, Ying-Liang Chou, Huey-Kang Sytwu, Mei-Cho Fang, Yuan-Yung Lin, Chao-Yin Kuo, Hsiao-Han Su, Chia-Lien Hung, Hang-Kang Chen, Chih-Hung Wang

**Affiliations:** ^1^ National Defense Medical Center, Graduate Institute of Medical Sciences, Taipei, Taiwan; ^2^ Department of Otolaryngology-Head and Neck Surgery, Tri-Service General Hospital, National Defense Medical Center, Taipei, Taiwan; ^3^ Department of Otorhinolaryngology, Taichung Armed Forces General Hospital, Taichung, Taiwan; ^4^ National Defense Medical Center, Graduate Institute of Medical Sciences, Taipei, Taiwan; ^5^ National Institute of Infectious Diseases and Vaccinology, National Health Research Institutes, Zhunan Town, Miaoli County, Taiwan; ^6^ Laboratory Animal Center, National Defense Medical Center, Taipei, Taiwan

**Keywords:** ultrasound microbubble (USMB), round window membrane (RWM), gold nanoparticle (AuNP), permeability, tight junction, inner ear, drug delivery, gene expression

## Abstract

The application of ultrasound microbubbles (USMBs) enhances the permeability of the round window membrane (RWM) and improves drug delivery to the inner ear. In this study, we investigated the efficiency of USMB-aided delivery of chitosan-coated gold nanoparticles (CS-AuNPs) and the mechanism of USMB-mediated enhancement of RMW permeability. We exposed mouse inner ears to USMBs at an intensity of 2 W/cm^2^ and then filled the tympanic bulla with CS-AuNPs or fluorescein isothiocyanate-decorated CS-AuNPs (FITC-CS-AuNPs). The membrane uptake of FITC-CS-AuNPs and their depth of permeation into the three-layer structure of the RWM, with or without prior USMB treatment, were visualized by z-stack confocal laser scanning microscopy. Ultrastructural changes in the RWM due to USMB-mediated cavitation appeared as sunburn-like peeling and various degrees of depression in the RWM surface, with pore-like openings forming in the outer epithelium. This disruption of the outer epithelium was paralleled by a transient reduction in tight junction (TJ)-associated protein levels in the RWM and an enhanced delivery of FITC-CS-AuNPs into the RWM. Without prior USMB exposure, the treatment with CS-AuNPs also caused a noticeable reduction in TJ proteins of the RWM. Our findings indicated that the combined treatment with USMBs and CS-AuNPs represents a promising and efficient drug and gene delivery vehicle for a *trans*-RWM approach for inner ear therapy. The outer epithelial layer of the RWM plays a decisive role in controlling the transmembrane transport of substances such as CS-AuNPs following the administration of USMBs. Most importantly, the enhanced permeation of AuNPs involved the transient disruption of the TJ-created paracellular barrier in the outer epithelium of the RWM.

## Introduction

Therapeutic approaches for the treatment of inner ear disorders include systemic delivery, intratympanic injection (IT), or intracochlear administration of drugs. At present, systemic drug delivery is regarded as the first-line approach for inner ear disorders (Mittal et al., 2019), despite drug delivery into the inner ear through the bloodstream being difficult and nondiscretionary. A systemically delivered drug must be able to cross the blood–labyrinth barrier between the inner ear and the surrounding vessels and tissues. However, many therapeutic agents cannot cross this barrier and remain in systemic circulation; thus, an adequate therapeutic concentration is not achieved in the inner ear (Inamura and Salt, 1992; Mittal et al., 2019). Advanced strategies are therefore imperative for more efficient drug delivery in the clinical setting.

The use of IT through the round window membrane (RWM) for drug administration to the inner ear has become popular in clinics. Examples include the treatment of Ménière’s disease with gentamicin and the treatment of sudden sensorineural hearing loss with steroids (Hoffer et al., 2006; Patel et al., 2016; El Sabbagh et al., 2017). However, this approach relies on the residence time of the drugs with the RWM; therefore, clinical applications of IT medication may require repeated injections. Patients receiving such injections must avoid swallowing, position their head slightly lower than the body, or lie in a supine position with the head turned to the contralateral side for some length of time (Shih et al., 2019). Furthermore, drug delivery through passive transportation, such as diffusion through the RWM, may be insufficient because the transfer of agents with a high molecular weight, such as peptide-protein drugs or genes, via the RWM route may be limited.

One approach that has potential for improving the transfer of drugs through the RWM is the use of nanomaterials, such as nanoparticles (NPs), which are now widely used in biomedical and industrial applications. Metallic NPs can be readily synthesized with biological rather than conventional chemical methods and have been applied in a wide range of research; for example, they have been used as catalysts, in pathogen detection, as antibacterial and anticancer agents, as drug or nucleic acid delivery vehicles, and as contrast agents for magnetic resonance imaging (Hong and Nam, 2014; Aouidat et al., 2019; Bao and Lan, 2019; Pissuwan et al., 2020). Various metals have been tested for this purpose, including Cu, Hg, Ag, Pt, and Au; gold nanoparticles (AuNPs) are the most stable and are now widely employed across the medical field (Hu et al., 2020).

The characteristics of AuNPs include high chemical and physical stability as well as straightforward functionalization with biologically active organic molecules or atoms; these properties are responsible for the excellent biocompatibility of AuNPs in both therapeutics and imaging applications, achieving the so-called theranostic purposes (Mittal et al., 2019; Pissuwan et al., 2020). The mechanism of AuNP-based drug delivery systems depends on nonspecific cellular endocytosis and subsequent release of the transported materials (Bannunah et al., 2014). The efficiency of AuNP delivery has been enhanced through modification of the chemisorption and electrostatic interactions between drugs and AuNPs to avoid drug degradation during endocytosis (Hong and Nam, 2014). AuNPs can be directly reduced and stabilized by chitosan (CS) to form a positively charged CS-AuNP core (Guo et al., 2010). The surface chemistry can be further altered with functional groups and ligands to target delivery of the decorated CS-AuNPs to a specific site of interest. In the case of the inner ear, various compositions of NPs have been tested; for example, poly (lactic-co-glycolic acid) NPs (Tamura et al., 2005); magnetic NPs; lipid nanocapsules (Zou et al., 2008); liposome, polymersome, and hydroxyapatite NPs; and silica NPs (Pyykkö et al., 2016). However, most of these NPs seldom reach the cochlear partition, and the release of a drug or gene of interest at the target site within the cochlea has not yet been demonstrated. Moreover, few studies have proposed AuNPs as a potential solution for inner ear theranostic applications (Kayyali et al., 2017).

Local RWM administration of AuNPs may be a superior protocol because this is a relatively noninvasive and accessible approach compared with opening the cochlear bony shell in cochleostomy. However, the RWM still poses a barrier to NP entry because of its three-layer sandwich-like structure. We previously used animal models to reveal that the use of ultrasound (US) microbubbles (MBs) can enhance the permeability of the RWM, resulting in a 38-fold enhancement of diffusion into the inner ear (Shih et al., 2013; Shih et al., 2019). As for the mechanism of enhanced permeability, it may involve a disruption of the continuity of the outer RWM epithelial layer, which controls the transmembrane transport of various substances (Lin et al., 2019). Recently, USMB cavitation was found to change the structure of tight junctions (TJs) and to reduce claudin-1 expression in RWM epithelial cells (Shang et al., 2015), suggesting that loss of the TJ-related primary barrier may play a role in enhanced diffusion.

The aim of the present study was to examine the use of CS-AuNPs as potential carriers for drug delivery to the inner ear. In particular, we investigated whether the use of USMBs would enhance the delivery of CS-AuNPs to the inner ear and whether changes in TJs were involved.

## Materials and Methods

### Animals and Study Design

**FIGURE 1:**
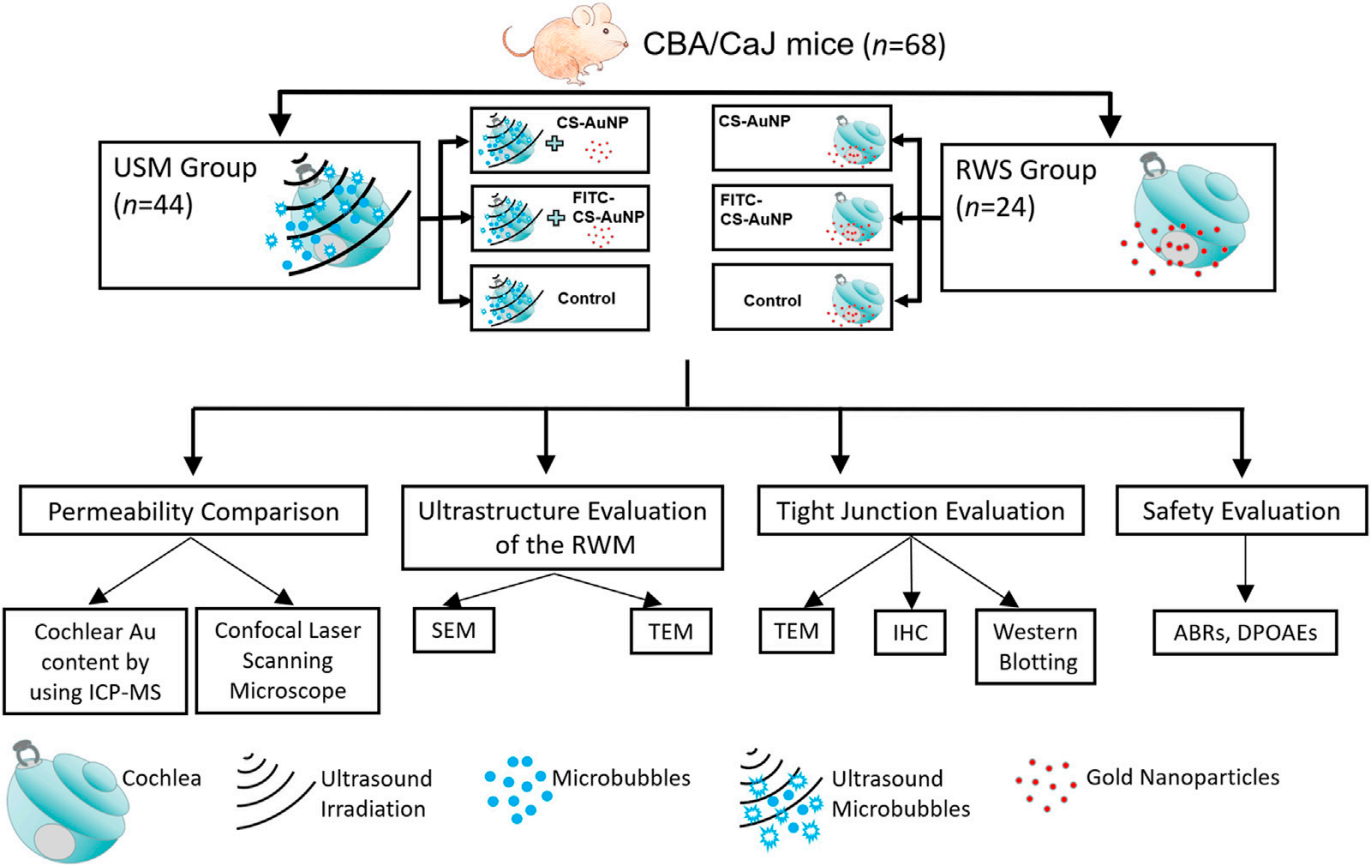
Schematic of this study. USM, ultrasound microbubble treatment; RWS, round window membrane soaking treatment; RWM, round window membrane; CS-AuNP, chitosan-coated gold nanoparticle; FITC, fluorescein thiocyanate; ICP-MS, inductively coupled plasma mass spectrometry; SEM, scanning electron microscopy; TEM, transmission electron microscopy; IHC, immunohistochemistry; ABRs, auditory brainstem responses; DPOAEs, distortion-product otoacoustic emissions.

Sixty-eight CBA/CaJ mice (8–10 weeks old, 20–25 g, purchased from The National Laboratory Animal Center, Taiwan) with a normal suprathreshold startle response (Preyer’s reflex) were allocated randomly to two groups (Figure 1). In the first group (the USMB group [USM]), the tympanic bulla was filled with 50 μl of MBs, which was followed by three consecutive 1-min US exposures. After each 1-min exposure, the used MBs were replaced with fresh MBs. Following the final USMB exposure, the MBs were removed, and the bulla was washed with 0.9% saline and then filled with 50 μl of CS-capped gold nanoparticles (i.e., CS-AuNPs) or fluorescein thiocyanate (FITC)-decorated CS-AuNPs (FITC-CS-AuNPs). In the second group, the RWM soaking (RWS) group, human clinical IT administration procedures were emulated. The tympanic bulla was filled with 50 μl of CS-AuNP conjugates. After 30 or 60 min, the animals were subjected to various hearing experiments. CS-AuNPs and FITC-CS-AuNPs were purchased from Tripod Nano Technology Co., Ltd. (Taoyuan, Taiwan).

### Cell Culture

HEI-OC1 cells, an auditory hair cell line that retains cell division activity (Kalinec et al., 2003), were kindly donated by Dr. Federico Kalinec (House Ear Institute, Los Angeles, CA, United States). HEI-OC1 cells were cultured in Dulbecco’s modified Eagle’s medium (Gibco, Grand Island, NY, United States) containing 10% fetal bovine serum (Biological Industries, Kibbutz Beit-Haemek, Israel) without antibiotics under permissive conditions (33°C and 10% CO_2_).

### Cell Viability for Different Ultrasound Power Densities

In total, 3 × 10^5^ cells/well were seeded into a 24-well cell culture plate and grown for 24 h. The culture medium was then replaced with 4 × 10^7^/200 μl MBs and sonicated by US at 1 MHz and various power densities (1, 2, and 3 W/cm^2^) for three consecutive 1-min exposures. The well plate was subsequently rinsed three times with phosphate buffered saline (PBS), after which the culture medium was replaced and the cells were cultured for a further 24 h. A cell proliferation reagent (WST-1; Roche Diagnostics GmbH, Mannheim, Germany) was added at 16  μl/well according to the manufacturer’s instructions and cultured for 4 h. The survival rate was quantified according to the 450-nm absorbance values measured using a hybrid multimode microplate reader (Synergy 2, BioTek Instruments, Winooski, VT, United States).

### Cytotoxicity Assay for Chitosan-Coated Gold Nanoparticles

HEI-OC1 cells were added to a 24-well cell culture plate and treated with USMBs at 1, 2, or 3 W/cm^2^. The cells were subsequently incubated with various concentrations of CS-AuNPs for 2 h and then evaluated for viability with the WST-1 assay.

### Microbubble Preparation and Ultrasound Exposure

As described in our previous study (Lin et al., 2019), SonoVue (Bracco, Milano, Italy) phospholipid MBs were freshly reconstituted prior to use by mixing the lyophilizate with 5 ml of 0.9% saline to form a suspension containing 2–5 × 10^8^ bubbles/mL, with a mean diameter of 5.7 μm. A US device (ST 2000 V, Nepa Gene, Chiba, Japan) equipped with a 6-mm diameter transducer was used for irradiation. The optimal US exposure settings were those determined in our previous report (Shih et al., 2019). Briefly, the mode was set as follows: frequency, 1 MHz; burst rate, 250 Hz; burst duration, 2 ms; acoustic intensity, 2 W/cm^2^ (mechanical index [MI] = 0.207) for three consecutive 1-min courses, and a 50% duty cycle. The transducer was positioned at the level of the mastoid bone with an opened tympanic bulla, which was at least 5 mm away from the RWM.

### Surgery

**FIGURE 2:**
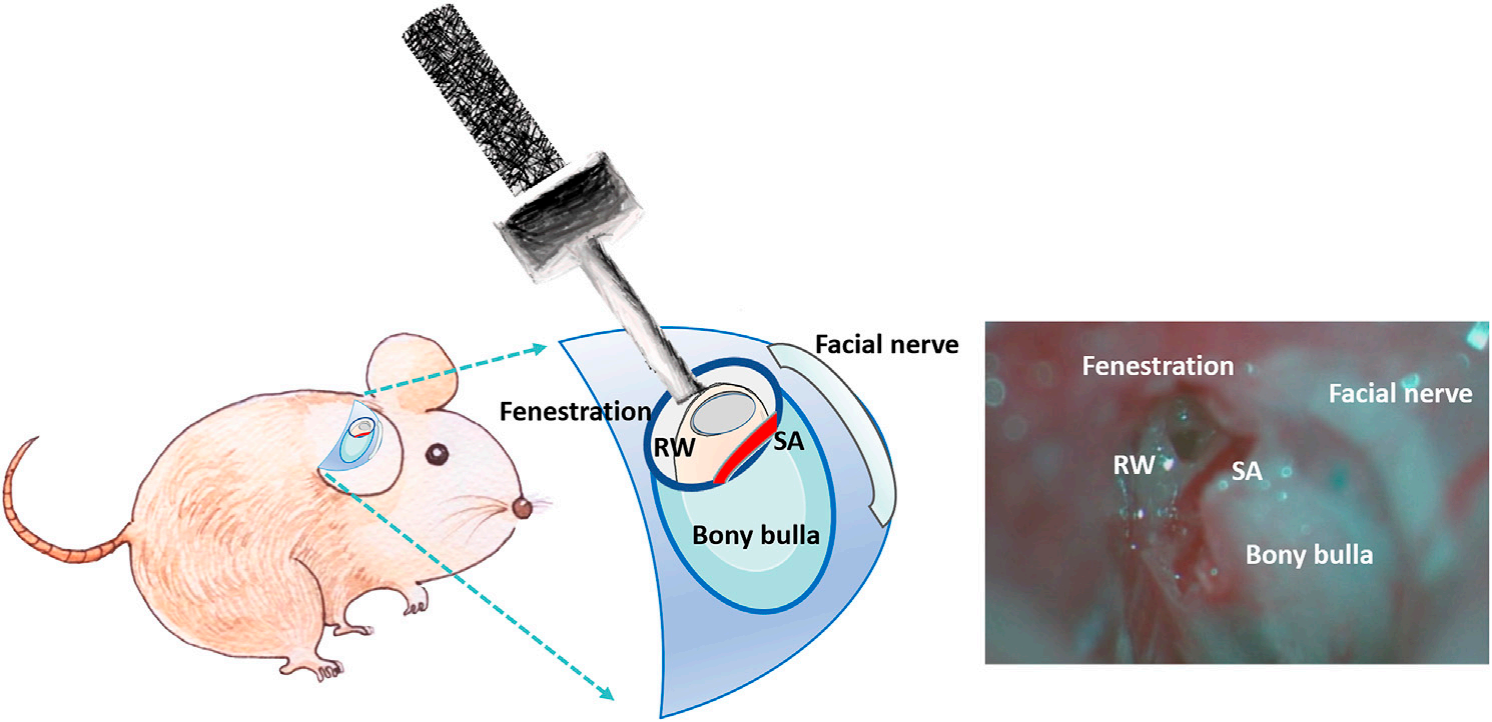
Schematic of the procedure for *in vivo* application of USMBs in the opened tympanic cavity.

Mice were anesthetized through intraperitoneal injection of a mixture of xylazine (16 mg/kg; Rompun; Bayer, Leverkusen, Germany) and ketamine (100 mg/kg; Imalgene; Merial, Lyon, France). All animals could spontaneously breathe room air and were placed on an electric heating pad. After we made a small transverse postauricular incision, we bluntly dissected the soft tissues to expose the tympanic bulla. We created a 5-mm-diameter fenestration in the tympanic bulla by drilling to expose the cochlea and RWM under an operating microscope (F-170; Carl Zeiss, Germany). US irradiation was then applied to the MBs filling the bulla (around 50–60 μl) through the bony fenestration in the USM group (Figure 2). For the RWS group, CS-AuNPs or FITC-CS-AuNPs were filled into the bulla and retained for the indicated duration without USMB exposure. The opened tympanic bulla was subsequently washed three times with 0.9% saline. For scanning electron microscopy (SEM), transmission electron microscopy (TEM), and confocal examination, the mice were sacrificed and the extracted temporal bones, including the cochleae, were fixed in various fixative solutions. For later auditory brainstem response (ABR) evaluation, the incision wound was closed with 5–0 nylon sutures.

### Lipopolysaccharides Treatment

One group of animals was treated with lipopolysaccharides (LPS), which are known to reduce the levels of TJ proteins. The animals in this LPS control group had their tympanic bulla filled with 50 μl of an emulsion of LPS in 0.9% sodium chloride (LPS, 5 mg/ml, Sigma, United States). After 48 h, the RWMs were dissected and prepared for the western blot analysis of TJ-related ZO-1 and occludin proteins.

### Immunohistochemistry

Animals were euthanized with an overdose of sodium pentobarbital and decapitated. Temporal bones were quickly removed, and the cochleae were treated with intrascalar perfusion with 4% paraformaldehyde in PBS (ChemCruz, Santa Cruz Biotechnology, Inc., Dallas, TX). Cochleae were fixed in 4% paraformaldehyde in PBS for 1 h at room temperature (RT) and then rinsed with PBS. The RWM was carefully dissected under a dissecting microscope and immersed in the same fixative at 4°C overnight. Postfixed RWMs were permeabilized using 0.1% Triton X-100 (Sigma) in PBS for 40 min at RT and then rinsed thoroughly in PBS. Tissues were incubated with primary mouse monoclonal anti-ZO1 antibodies (1:100; Thermo Fisher Scientific, Waltham, MA) and rabbit monoclonal antioccludin antibodies (1:100; Abcam Inc., Cambridge, United Kingdom). After three 10 min washes with PBS, tissues were incubated with Alexa Fluor 488–labeled donkey antimouse antibodies and 555-labeled goat antirabbit antibodies (1:500; Thermo Fisher Scientific) for 2 h at RT. Following incubation with Alexa Fluor 633–labeled phalloidin (1:100; A22284, Thermo Fisher Scientific) for 30 min, samples were mounted in 4,6-diamidino-2-phenylindole (DAPI) Fluoromount-G mounting medium and covered with a coverslip. The fluorescence images were obtained with a confocal laser scanning microscope (Zeiss LSM 880, Carl Zeiss).

### Scanning Electron Microscopy

In brief, the removed cochleae were placed in electron microscopy fixative (0.1% sodium cacodylate–buffered 2.5% glutaraldehyde with 2% paraformaldehyde) overnight at 4°C and then given three 10-min washes with cold PBS (0.1 M, pH 7.4). For RWM preparation, the specimens were dissected and trimmed, leaving the RWM tissue intact (Lin et al., 2019). Samples were washed with three changes of 0.1 M cacodylate buffer containing 7% sucrose. After postfixing in 1% osmium tetroxide (OsO_4_; Electron Microscopy Science [EMS], Hatfield, PA, United States) and 1% thiocarbohydrizide (TCH; EMS) for 2 h, the samples were again given three 15-min washes with 0.1 M cacodylate buffer containing 7% sucrose. The specimens were dehydrated through a graded ethanol series (35% to absolute ethanol) at 10-min intervals and then finished in a critical point dryer. We viewed and photographed the processed specimens by using an SU3500 scanning electron microscope (Hitachi, Tokyo, Japan) at 15 kV.

### Transmission Electron Microscopy

Consistent with the procedures for SEM, the cochleae were fixed overnight at 4°C, washed with cold PBS, and dissected and trimmed to leave only the intact RWM soft tissue. The whole RWM was postfixed in 1% OsO_4_ for 2 h, given three 15-min washes with 0.1 M PBS, dehydrated in an ethanol series, infiltrated with Spurr’s resin, and polymerized. The embedded samples were sectioned with an ultramicrotome (Leica EM UC7) at a 90-nm thickness. Images were obtained using a transmission electron microscope (Philips/FEI Tecnai 20 G2 S-Twin).

### Confocal Laser Scanning Microscopy

HEI-OC1 cells were transferred to a sterilized, removable 8-well chamber (ibidi GmbH, Martinsried, Germany) and treated with USMBs at 1, 2, or 3 W/cm^2^. The cells were then incubated for 24 h with various concentrations of FITC-CS-AuNP. This was followed by fixation with 4% paraformaldehyde, rinsing with PBS, mounting in DAPI Fluoromount-G mounting medium, and covering with a coverslip. The fluorescence images were obtained with a confocal laser scanning microscope (Zeiss LSM 880, Carl Zeiss) in the z-stack scanning mode using a ×63 oil objective. For the RWM confocal laser scanning microscopy (CLSM) examination, the mouse cochleae were first treated with 2 W/cm^2^ USMBs, subsequently incubated with various concentrations of FITC-CS-AuNPs, and then washed and fixed in 4% paraformaldehyde. The RWM was dissected from the bulla, mounted in DAPI Fluoromount-G mounting medium, and covered with a coverslip. The fluorescence images were obtained in the z-stack scanning mode using a ×40 objective with a step size of 1 μm. Both scanning images of the x–z and y–z plane obtained from the sample were used to visualize the distribution of fluorescence across the whole thickness of three-layered RWM. The images were recorded using ZEN 2012 (blue edition) software. For the quantification of fluorescence, intensity images were acquired and analyzed using ImageJ (version 1.51, United States National Institutes of Health).

### Western Blotting

Proteins in aliquots of whole RWM homogenates were separated on mPAGE 4–12% Bis–Tris gels (Merck Millipore, Burlington, MA, United States), transferred to polyvinylidene difluoride membranes (Merck Millipore, Billerica, MA), blocked with blocking buffer (BlockPRO, Visual Protein Biotechnology Corporation, Taiwan), and probed with the indicated primary antibody at 4°C overnight. After we washed the membranes three times with Tris-buffered saline with 0.1% Tween 20 (TBST), they were incubated with antimouse or antirabbit horseradish peroxidase–linked whole antibody (1:10,000; GE Healthcare, Chicago, IL, United States) for 1 h at RT and again washed with TBST. The immunoreactive bands were stained using a light-emitting nonradioactive method (ECL; Merck Millipore, Burlington, MA). The specific primary antibodies included mouse monoclonal anti-ZO1 antibody (1:500; Thermo Fisher Scientific), rabbit monoclonal antioccludin antibody (1:1,000; Abcam Inc., Cambridge, United Kingdom), and mouse monoclonal antiactin antibody (1:1,000; Merck Millipore, Burlington, MA). Images were acquired and analyzed using ImageJ for band intensity quantification.

### Auditory Brainstem Response Recording

The animals’ auditory function was evaluated by recording their ABRs. Mice were anesthetized and kept warm with a heating pad in a sound-attenuating chamber. Subcutaneous needle electrodes were inserted at the vertex (positive electrode), below the pinna of the ear (negative electrode), and at the back (ground electrode). Specific stimuli (clicks and 8-, 16-, and 32-kHz tone bursts) were generated using SigGen software (Tucker-Davis Technologies, Alachua, FL, United States) and delivered through an earphone inserted into the external auditory canal. The average responses from 1,024 stimuli for each frequency were obtained by reducing the sound intensity in 5-dB steps until reaching a threshold. The resulting ABR thresholds were defined as the lowest intensity at which a reproducible deflection in the evoked response trace could be recognized.

### Distortion-Product Otoacoustic Emission Measurements

The distortion-product otoacoustic emissions (DPOAEs) were measured at center frequencies (CFs) of 8, 16, 20, 24, and 32 kHz with a real-time signal processing system (Tucker-Davis Technologies, Gainesville, FL, United States), as described previously (Chen et al., 2018). Two simultaneously presented pure tones, F1 and F2, were calculated using the CF, where F1 was CF × 0.909 and F2 was CF × 1.09. This yielded a frequency of primary 1 (Tone 1) and primary 2 (Tone 2) geometrically centered on the CF. The two primary tones were presented at the same intensity (L1 = L2 = 65 dB) and at a frequency ratio (F2:F1) of 1:2. The primary tones produced by two separate speakers (EC1 close-field speakers; Tucker-Davis Technologies) were transmitted into each animal’s ear canal. DPOAE recordings were made with a low-noise microphone (ER 10B+; Etymotic Research, Elk Grove Village, IL, United States) and averaged 512 times at each frequency. The peak of the cubic difference distortion product (2F1–F2) at various CFs was accepted as a DPOAE if it was 3 dB above the noise floor. The difference was referred to as the signal-to-noise ratio (SNR).

### Quantitative Determination of Gold Nanoparticles Through Inductively Coupled Plasma Mass Spectrometry

The efficiency of AuNP entry into the cochleae was quantitatively analyzed by adding 3 ml of concentrated nitric acid (Ultrex II, J.T. Baker, MT, United States) to microwave digestion vessels containing 100 μl of AuNPs, which was followed by digestion in a CEM Mars five Microwave Accelerated Reaction System (CEM Corp, Matthews, NC, United States). The parameters of the microwaving cycle were as follows: power, 240 W; temperature, 180°C; pressure, 170 psi; and hold time, 15 min. Subsequently, the AuNPs were cooled to RT. The digested samples were diluted to 25 ml with double-distilled water and analyzed by inductively coupled plasma mass spectrometry (ICP-MS; XSeries 2 spectrometer; Thermo Fisher, Hemel Hempstead, United Kingdom). Typical normal mode conditions were employed: extraction voltage, −35 V; Rf Power, 510 W; focus voltage, 4.5 V; and nebulizer gas flow rate, 1.04 L/min. Dwell times were 10 ms, with 100 sweeps per replicate and five replicates per sample. The Au standard for ICP (1,000 mg/L Au Certipur, Merck KGaA, Darmstadt, Germany) was used for preparing working standard solutions [19]. The Au content (μg/mL of AuNPs), as determined through ICP-MS, was 0–100 ng/ml.

### Statistical Analysis

Statistical analysis was performed using a two-tailed Student’s *t* test for comparison of the means between two groups and the Kruskal–Wallis test and one-way analysis of variance (ANOVA) for multigroup comparisons, followed by Bonferroni post hoc tests (significance levels: *p < 0.05, **p < 0.01, ***p < 0.001). Data are expressed as means ± standard error of the mean.

## Results

### Cellular Uptake of Chitosan-Coated Gold Nanoparticles Was Increased Through Ultrasound Microbubbles Treatment

**FIGURE 3:**
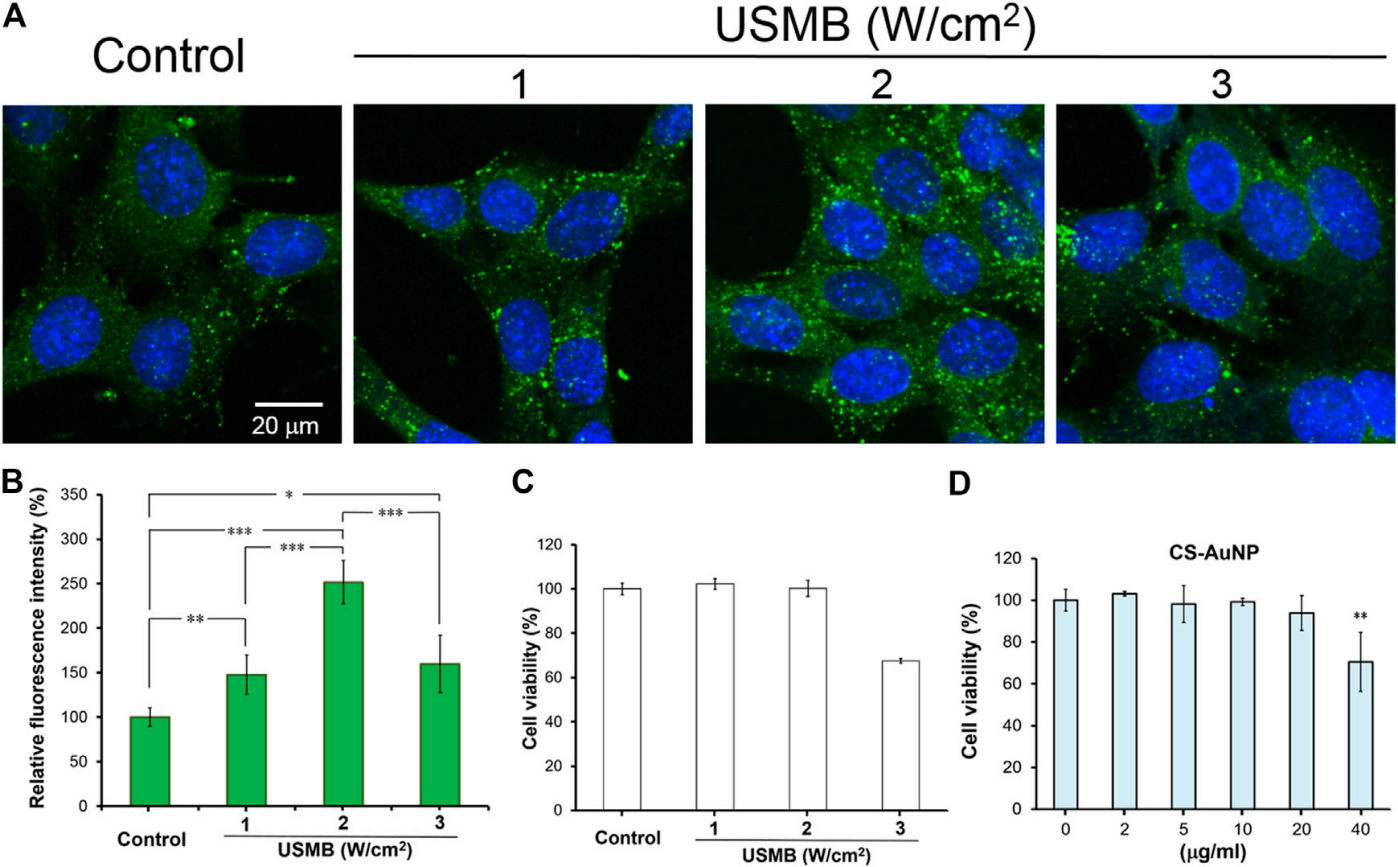
Comparison of the permeability difference of the HEI-OC1 cells following FITC-CS-AuNP treatments with or without prior USMB exposure. Fluorescence images of **(A)** HEI-OC1cells after 24-h incubation with FITC-CS-AuNPs at a concentration of 10 μg/ml. Prior to FITC-CS-AuNP treatment, three consecutive USMB exposures (1 min/exposure) at 1 MHz frequency and an acoustic intensity of 1, 2, or 3 W/cm^2^ were conducted. **(B)** Quantitative analysis of cellular uptake of FITC-CS-AuNPs by HEI-OC1 cells. **(C)** WST-1 assay of the HEI-OC1 cells after exposure to various intensities of US with MBs for 24 h. Control corresponds to cells without USMB treatment. **(D)** WST-1 assay of the HEI-OC1 cells after incubation with various concentrations of CS-AuNP for 24 h. Control corresponds to cells in the culture medium without CS-AuNPs. *p < 0.05, **p < 0.01, ***p < 0.001. USMB, ultrasound microbubble; CS-AuNPs, chitosan-coated gold nanoparticles.

Figures 3A,B reveal that uptake of CS-AuNPs by HEI-OC1 cells was significantly increased when cells were pretreated with three consecutive USMB exposures at all applied acoustic intensities. However, at 3 W/cm^2^, cell viability was compromised and reduced to 67.49 ± 1.04% in comparison with the control, as determined by the WST-1 assay (Figure 3C). Suitable NP concentrations for experiments were identified by treating cells with various concentrations of CS-AuNPs (2, 5, 10, 20, and 40 μg/ml). The highest concentration (40 μg/ml) caused a significant 30% cell loss (Figure 3D), indicating that cell viability decreased with increasing concentrations of AuNPs. Therefore, we selected CS-AuNPs at a concentration of 10 μg/ml and USMB treatment at a US intensity of 2 W/cm^2^ for subsequent cell uptake experiments.

### Effects of Ultrasound-Mediated Microbubble Cavitation and Sonoporation on the Outer Epithelial Layer of the Round Window Membrane

**FIGURE 4:**
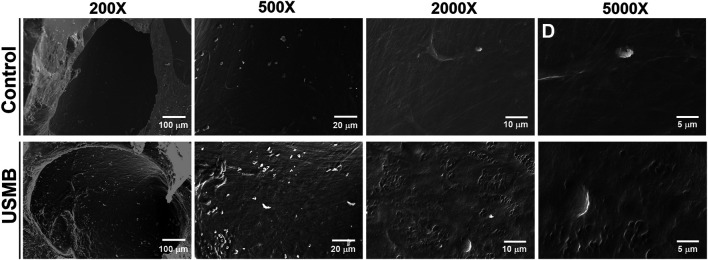
Scanning electron micrographs of the epithelial surface of the RWM under various magnifications. RWM samples in the USM group were immediately taken from the animal after three exposure courses of USMBs. Samples from animals after MB soaking of the tympanic bulla for the same period without US exposure served as controls. USMB, ultrasound microbubble.

**FIGURE 5:**
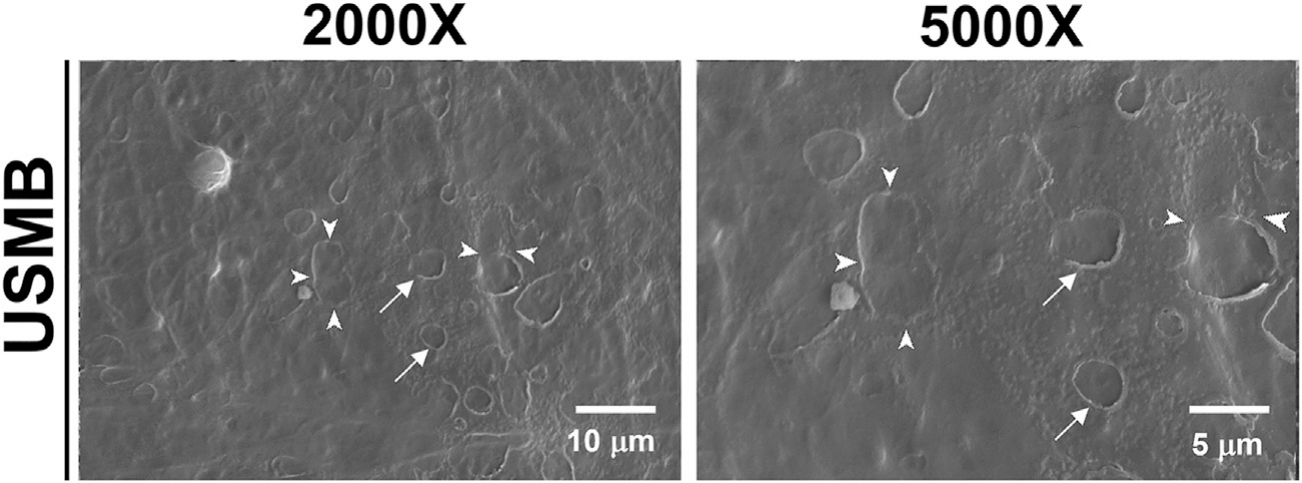
Scanning electron micrographs of the epithelial surface of the RWM. After USMB treatment, the epithelium exhibited sunburn-like peeling, either in separate circular shapes (arrows) or fused forms (arrowheads). USMB, ultrasound microbubble.

**FIGURE 6:**
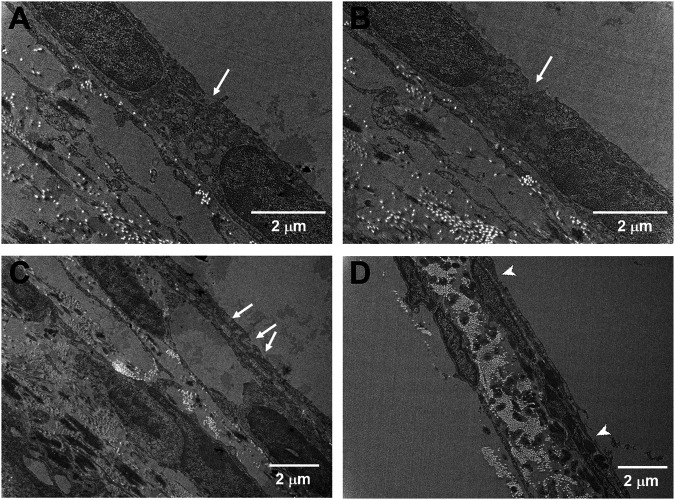
Cross-sectional TEM images of the USMB-treated RWM under different magnifications. Samples were taken immediately from the mice after three USMB treatments. **(A−C)** Pore-like openings with various depths and sizes (arrows) were distributed along the outer epithelium of the RWM. **(D)** Some areas of the membrane exhibited sunburn-like peeling over the outer epithelium (arrowheads).

We previously revealed that USMB-mediated cavitation caused the formation, to varying degrees, of heterogeneous pore-like openings on the RWM of guinea pigs and that these openings enhanced the permeability of the RWM (Lin et al., 2019). Using mice as an experimental model in this study, we conducted similar USMB exposures, using three consecutive 1-min courses and a 50% duty cycle (Lin et al., 2019), but we reduced the acoustic intensity to 2 W/cm^2^. As presented in Figure 4, the USMB group immediately after USMB treatment exhibited varying degrees of depressions on the RWM surface, with pore-like openings ranging from 0.1 to several microns and resembling pitting corrosion on metals. Sunburn-like peeling of the epithelia, either in separate or fused circular shapes, was also distributed on the surface of the USMB-exposed RMW (Figure 5). TEM confirmed the presence of pore-like openings of various depths and sizes (Figure 6). Unlike the ultrastructural changes observed in the RWM in guinea pigs, the mouse RWM exhibited no significant epithelial tears or ruptures after USMB treatment at an acoustic power intensity of 2 W/cm^2^.

**FIGURE 7:**
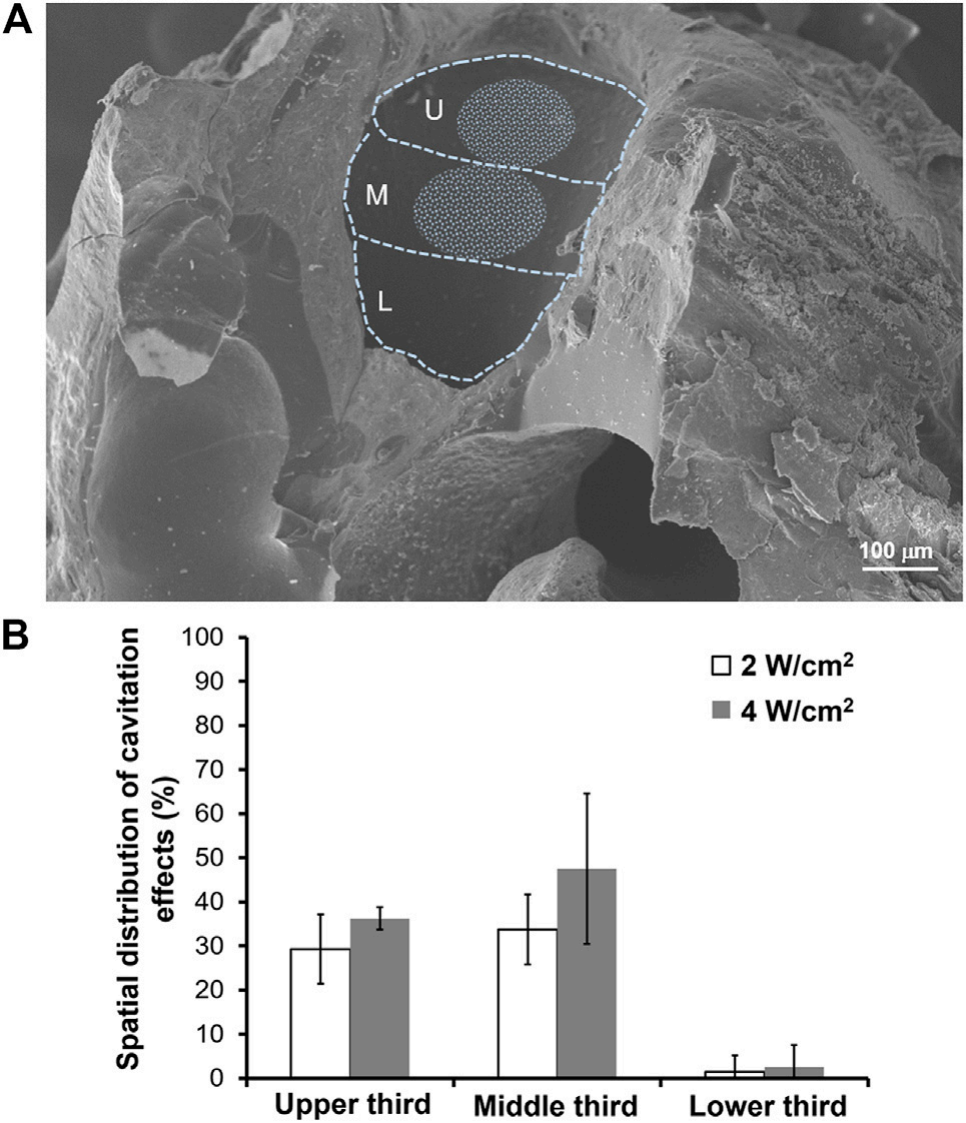
Cavitation effects area of the RWM following USMB treatment. **(A)** A representative SEM image of the left side of the RWM was divided into upper, middle, and lower third parts. The whitish circled area indicates the area where USMBs reacted at an acoustic intensity of 2 W/cm^2^. **(B)** Comparison of the proportion of the reaction area at acoustic intensities of 2 and 4 W/cm^2^. U = upper third; M = middle third; L = lower third. The results are expressed as the mean ± standard error of the mean, with *n* = 10 for each bar in the 2 W/cm^2^ group and *n* = 4 in the 4 W/cm^2^ group.

We also investigated the site on the RWM that was most affected by USMB-mediated cavitation and the possibility that the involved area changed with increasing acoustic power intensity. As displayed in Figure 7A, at a USMB treatment intensity of 2 W/cm^2^, the largest sonication effect was observed in the middle third of the RWM (33.75 ± 7.91%), followed by the upper third (29.29 ± 7.87%) and the lower third (1.43 ± 3.78%). When we increased the power density to 4 W/cm^2^, these reaction areas also increased (upper third: 36.25 ± 2.50%; middle third: 47.50 ± 17.08%; lower third: 2.50 ± 5.00%), but the differences were not statistically significant compared with the values obtained at 2 W/cm^2^ (Figure 7B). These findings suggest that USMB-mediated cavitation was focused on the middle and upper third regions of the RWM, which comprise approximately one-quarter of the whole area of the RWM.

### Ultrasound Microbubbles Treatment of the Round Window Membrane Facilitates the Penetration of Chitosan-Coated Gold Nanoparticles

**FIGURE 8:**
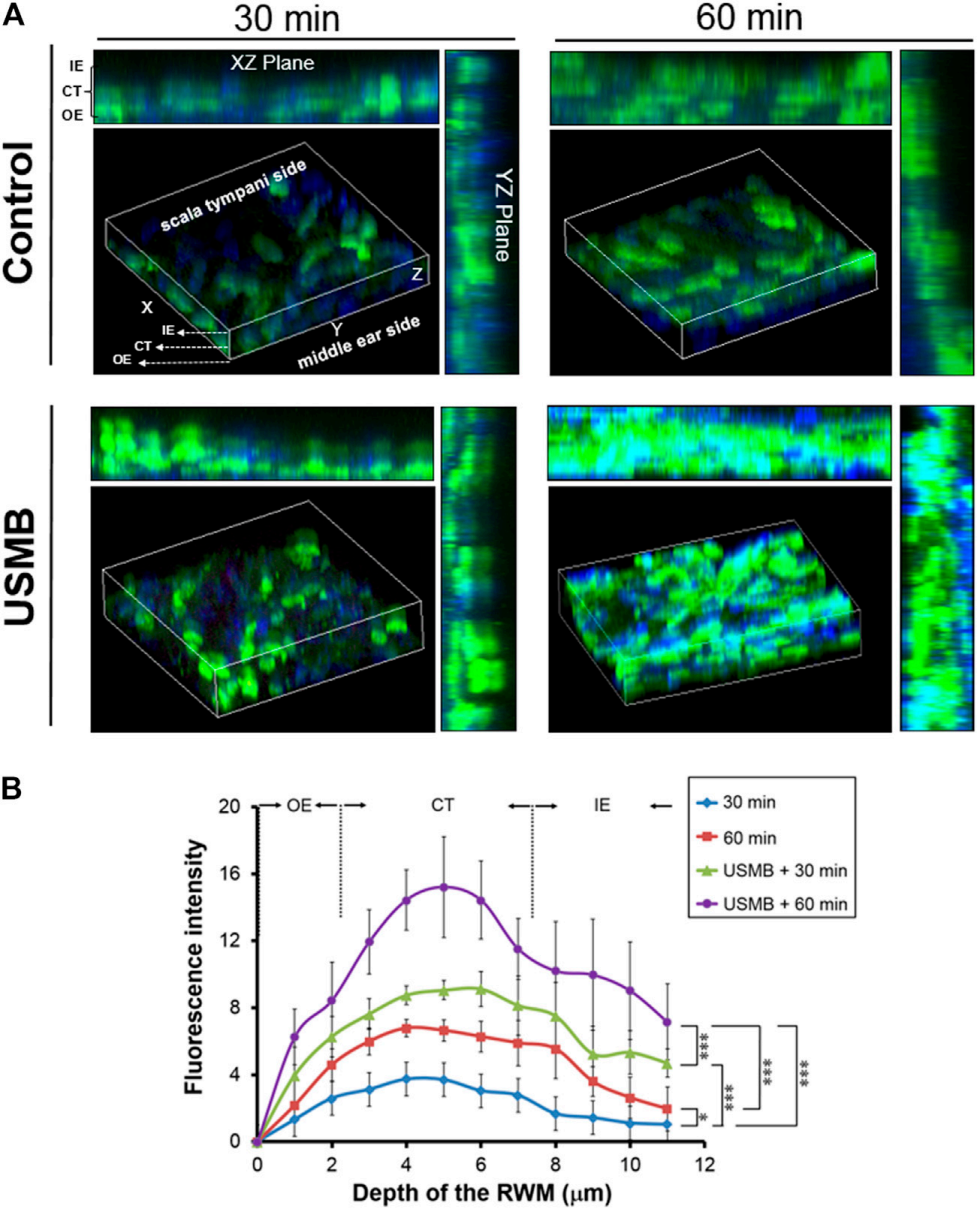
Series of confocal laser scanning microscope images of the RWM after FITC-CS-AuNP treatment with or without prior USMB exposure. **(A)** Three-dimensional reconstruction of the RWM by z-stack imaging was used to assess penetration of fluorescent FITC-CS-AuNPs through the three-layer membrane after treatment for 30 and 60 min. Control group = mouse tympanic bulla soaked with FITC-CS-AuNPs for 30 and 60 min. USMB group = mouse tympanic bulla exposed to USMB first, followed by soaking with FITC-CS-AuNPs for 30 and 60 min. **(B)** Quantitative analysis of the fluorescence intensity of z-stacking images. Values are expressed as mean ± standard error of the mean (*n* = 5). RWM, round window membrane; USMB, ultrasound microbubble. *p < 0.05, **p < 0.01, ***p < 0.001 (one-way ANOVA, with post hoc Bonferroni tests).

We previously reported that exposing the RWM to USMBs increased membrane permeability and could enhance drug delivery into the inner ear (Shih et al., 2013; Shih et al., 2019; Liao et al., 2020). Here, we investigated whether USMB treatment of the RWM could also facilitate the penetration of CS-AuNPs. As presented in Figure 8, USMB exposure enhanced the penetration of FITC-labeled CS-AuNPs through the RWM, as determined through CLSM. The RWM consists of three layers: the outer epithelium, middle connective tissue, and inner epithelium. Treatment with FITC-CS-AuNPs for 30 min resulted in the appearance of fluorescent CS-AuNPs in the inner epithelial layer in the USMB-treated group, whereas no penetration to the inner epithelial layer was observed in the control group not treated with USMBs (Figure 8A). Sixty minutes after USMB exposure, FITC-CS-AuNPs were noted across all three layers of the RWM in both groups; however, quantitative analysis revealed greater fluorescence intensity in the inner epithelial layer in the USMB-treated group than in the control (*p* < 0.001; Figure 8B). The middle connective tissue layer is the thickest of the three layers of the RWM and thus may accumulate most of the fluorescent FITC-CS-AuNPs. Quantitative comparison of the efficiency of FITC-CS-AuNPs passage through the three-layer membrane revealed greater penetration in the USMB-treated group than in the control group at both 30 min (*p* < 0.001, one-way ANOVA and Bonferroni post hoc test) and 60 min (*p* < 0.001, one-way ANOVA and Bonferroni post hoc test). These results suggest that USMB treatment of the RWM could promote AuNP penetration through the outer epithelium to the inner epithelial layer of the RWM.

### Mechanism of Ultrasound Microbubbles Treatment-Mediated Permeability Enhancement Involves a Transient Disruption of Tight Junction Integrity

**FIGURE 9:**
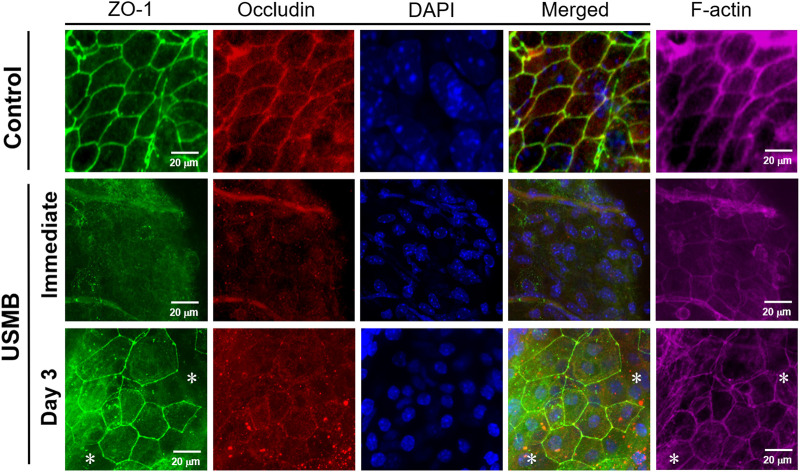
Immunohistochemical detection of the TJ-associated molecules ZO-1 (green), occludin (red), and F-actin (far-red) in the RWM immediately following and 3 days after USMB treatment. The membranes without USMB treatment served as controls. Nuclei were stained with DAPI (blue). Asterisks (*) indicate areas of the cell boundary staining positive for F-actin but negative for ZO-1. USMB, ultrasound microbubble.

The basis for the “enhanced permeability” of the RWM following USMB treatment is not completely understood, although we reported a disruption in the continuity of the outer RWM epithelial layer following USMB treatment in our previous study (Lin et al., 2019). Because the outer epithelium is the main barrier preventing the passage of substances into the RWM, we investigated the role of the TJs between the outer epithelial cells. As displayed in Figure 9, the expression of zonula occludens-1 (ZO-1), a TJ-specific submembranous protein, and the expression of occludin, a transmembrane protein that maintains TJ integrity, were clearly reduced, as indicated by the less intense staining of the USMB-treated RWM compared with the untreated control. Notably, the reduced staining intensity for ZO-1 and occludin at the cell boundaries was recovered 3 days after USMB treatment. This reassembly may only be partial because the cell–cell boundaries of epithelia from the RWM, where F-actin and ZO-1 are colocalized, showed clearly visible F-actin staining, but ZO-1 staining was absent (Figure 9, asterisks). F-actin protein has a known involvement in maintaining the integrity of the epithelial barrier and in cytoskeleton organization (Ivanov et al., 2010; Rodgers and Fanning, 2011). USMB treatment also reduced the expression of F-actin in the outer epithelium of the RWM when compared with its abundant expression in the untreated control group.

**FIGURE 10:**
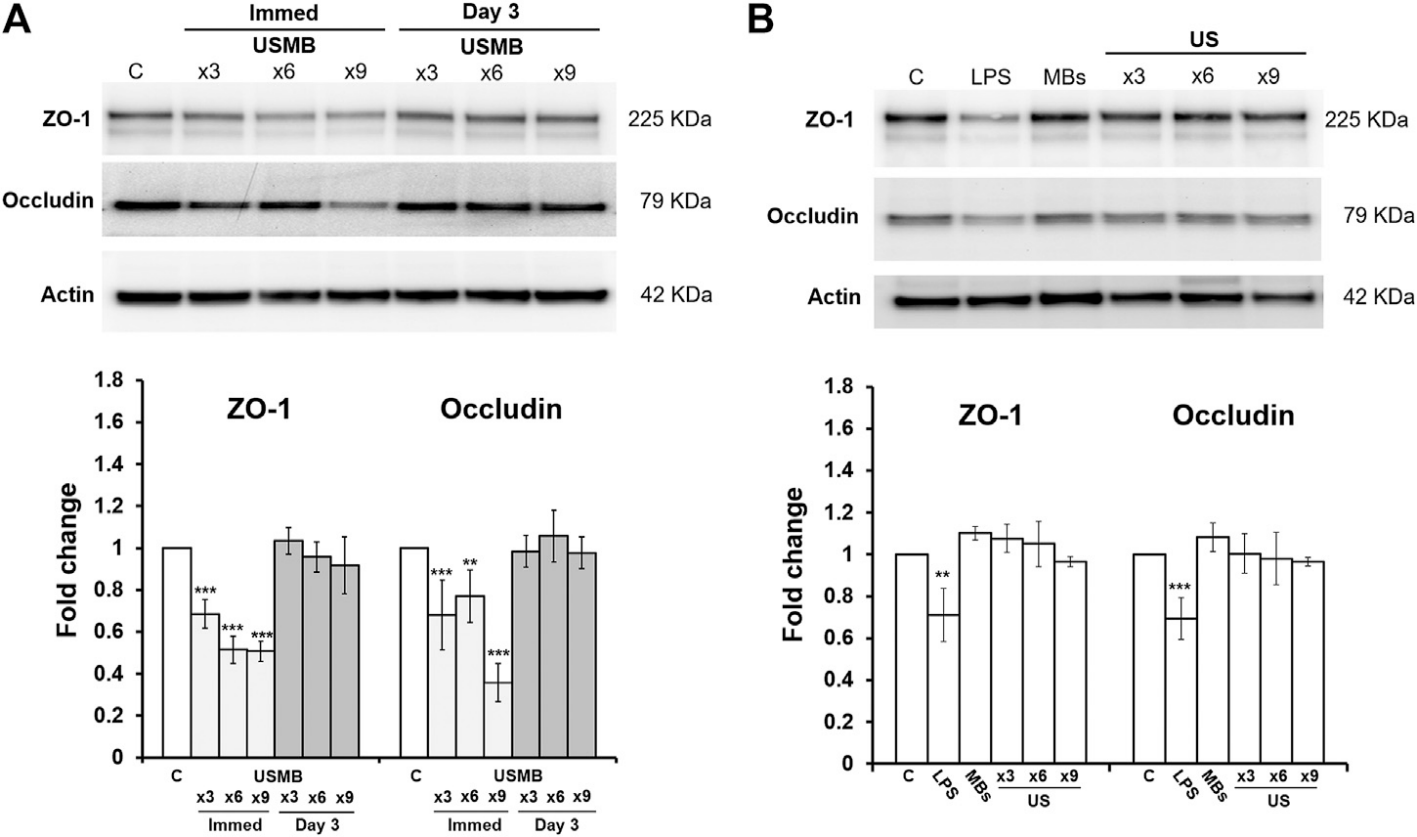
Representative western blot analysis of ZO-1 and occludin protein levels of the RWM **(upper panels)** and digital quantification **(lower panels)**. Untreated RWM served as a control and LPS-treated RWM served as a positive control for TJ protein reduction. **(A)** Both ZO-1 and occludin protein amounts in the RWM were significantly reduced immediately after various courses of USMB exposure, but the levels recovered 3 days later (*n* = 4 for each bar). **(B)** Both ZO-1 and occludin protein amounts in the RWM revealed no significant difference in untreated RWM, RWM soaked with MBs but without US exposure, or RWM given various courses of US exposure but without MBs (*n* = 4 for each bar). The results are expressed as the mean ± standard error of the mean for the fold change in protein expression level relative to control. **p < 0.01; ***p < 0.001; C, control; LPS, lipopolysaccharides; MBs, microbubbles; Immed, immediate; US, ultrasound; USMB, ultrasound microbubble.

Western blot analysis also revealed reductions in both ZO-1 and occludin protein levels immediately after USMB treatment for three-, six-, and nine-course exposures. The loss of protein was associated with the sonication exposure, implying that the degree of tissue damage was correlated with cavitation intensity. However, both proteins exhibited recovery 3 days later (Figure 10A), in agreement with the immunohistochemistry findings following a three-course USMB treatment (Figure 9). These data indicate that USMB treatment caused a transient loss of TJ-associated proteins in the RWM.

By contrast, applying US in the absence of MBs or soaking the RWM with MBs in the absence of US exposure did not change the amounts of TJ proteins in the RWM (Figure 10B). This suggests that the integrity of the TJ and TJ proteins in the RWM are only vulnerable to US exposure when combined with MB treatment, in agreement with our previous findings (Shih et al., 2013).

**FIGURE 11:**
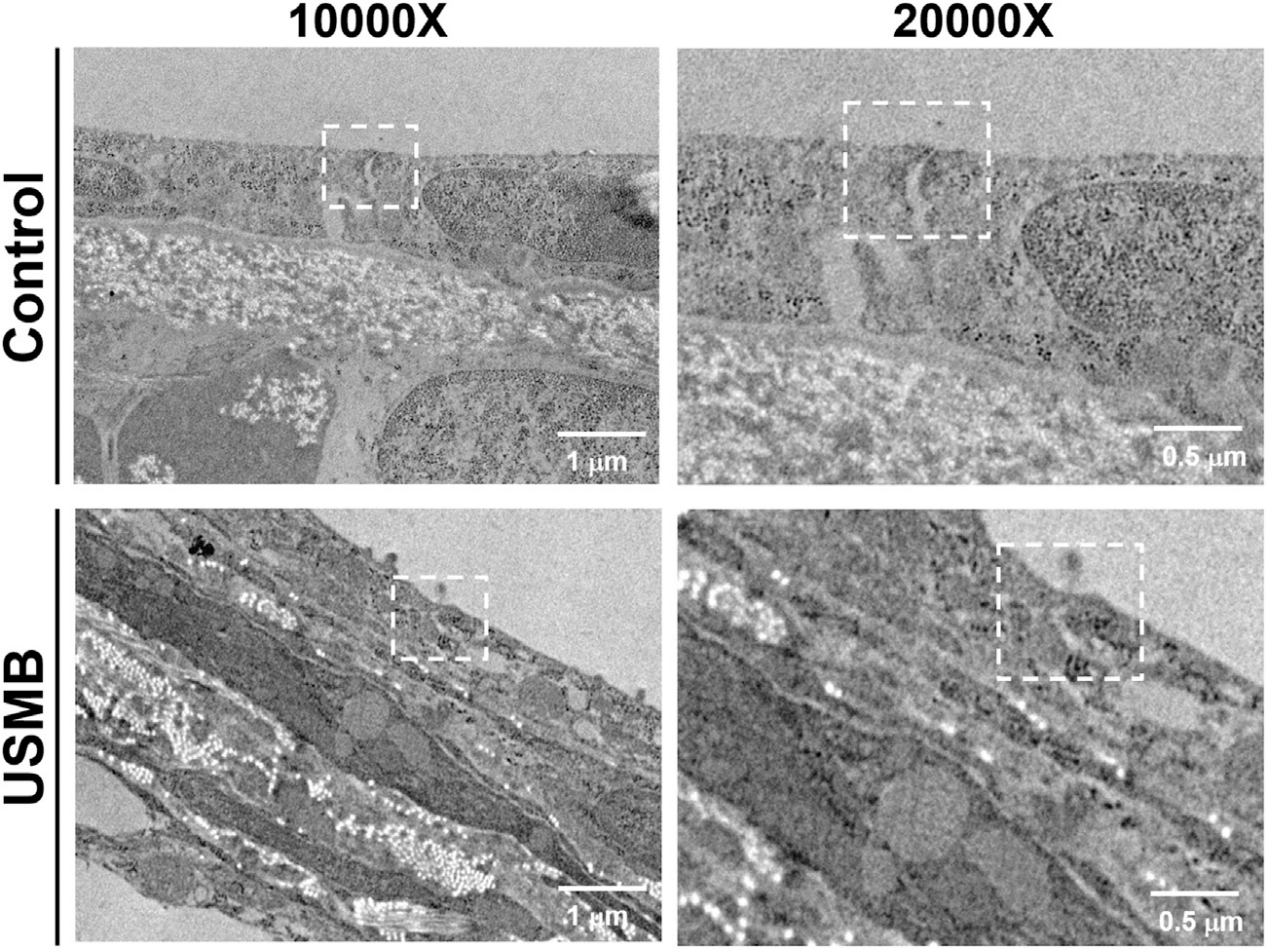
Transmission electron micrographs of the TJ region in the outer epithelium of the RWM with **(lower panels)** or without prior USMB exposure **(upper panels)**. Cell membranes of adjacent cells in close apposition in a TJ of the control group exhibited more significant accumulation of perijunctional actin protein complexes (white squares). This protein complex was absent in the TJ region of adjacent cells after USMB treatment. USMB, group receiving the ultrasound microbubble treatment; Control, mouse group treated with microbubbles but without ultrasound exposure.

TEM examination of the ultrastructural changes in the RWM revealed that the outer epithelium in the unexposed control animals had a normal architecture. The TJs between adjacent flat outer epithelial cells exhibited typical features of multiprotein junctional complexes along the apical region (Figure 11 **)**. USMB treatment disrupted the dense perijunctional protein complexes, indicating that the enhanced permeability of the RWM after USMB treatment involved a disturbance of the TJs in the outer epithelium of the RWM.

### Ultrasound Microbubbles Treatment-Mediated Cavitation Promotes the Uptake of Gold Nanoparticles Into the Inner Ear Through the Round Window Membrane

**FIGURE 12:**
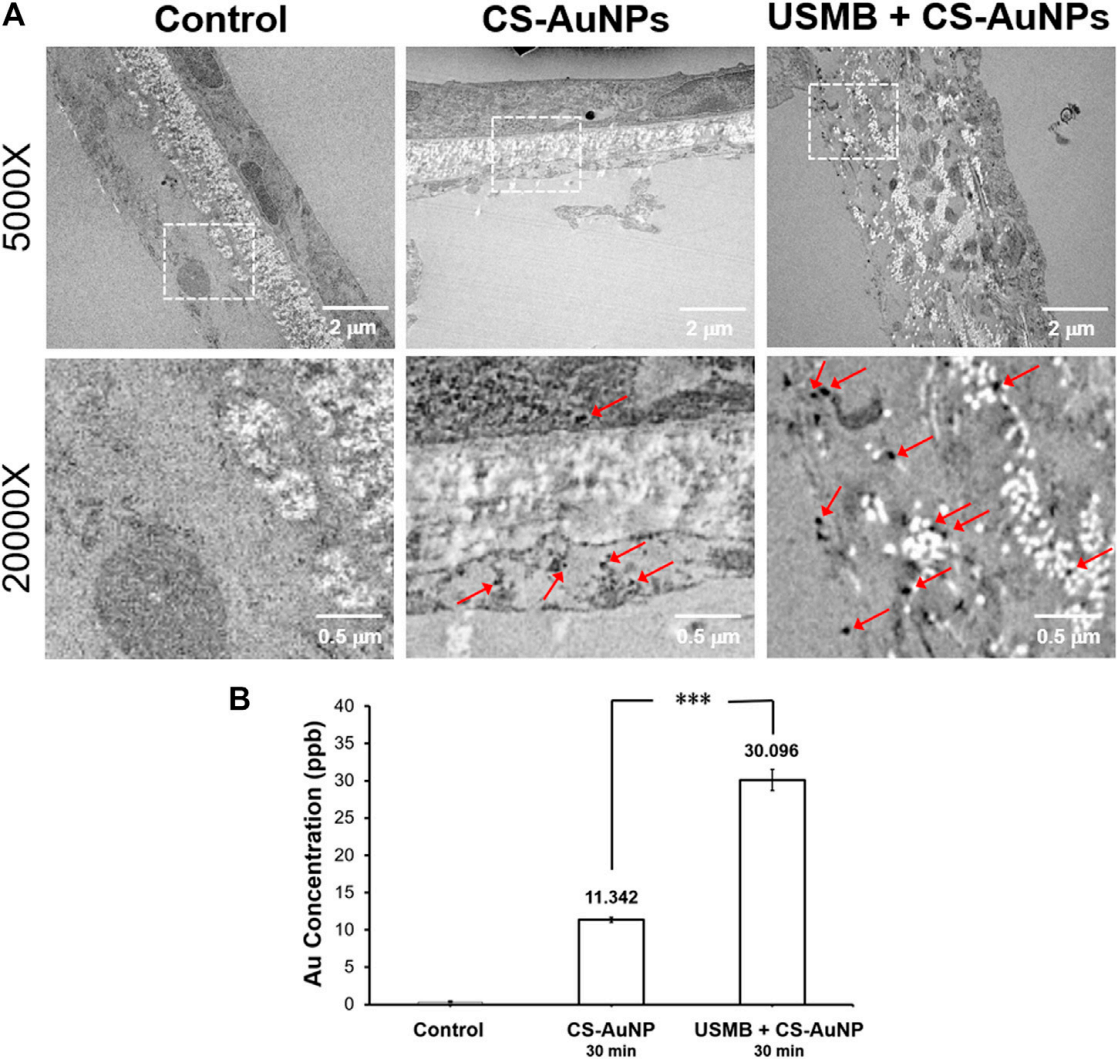
**(A)** Transmission electron micrographs of a CS-AuNP-treated RWM with or without preceding USMB exposure **(upper panels)**. The lower panels reveal the magnification of the square region in the upper panels, where multiple AuNPs can be observed (arrows). **(B)** Comparison of the delivered Au concentration in the inner ear of the USM and RWS groups by ICP-MS analysis. The results are expressed as the mean ± standard error of the mean, with *n* = 4 for each bar. ***p < 0.001; CS-AuNP, chitosan-coated gold nanoparticles; USMB, ultrasound microbubble.

We also examined whether USMB-mediated permeability changes in the RWM can promote the delivery of AuNPs into the inner ear. Using mice as an experimental model, the tympanic bulla containing the cochlea and the round window were soaked with CS-AuNPs for 30 min, with or without prior USMB treatment. TEM examination of the RWM revealed that the AuNPs had passed through the outer epithelium and middle connective tissue layer and had reached the inner epithelium (Figure 12A, arrows). The amount of CS-AuNPs was greater in the inner epithelium of the USM group than in the control group, in agreement with the results shown in Figure 8. The delivery of CS-AuNPs into the inner ear was also significantly higher in the USM group than in the control group (*p* < 0.001, Figure 12B; Supplementary Figure S1). These data indicate that although CS-AuNPs with a small particle size can pass through cells of the RWM, application of USMB can further enhance this cellular uptake of CS-AuNPs and deliver them into the inner ear.

### Effects of Chitosan-Coated Gold Nanoparticles on the Expression of ZO-1 and Occludin Proteins in the Round Window Membrane

**FIGURE 13:**
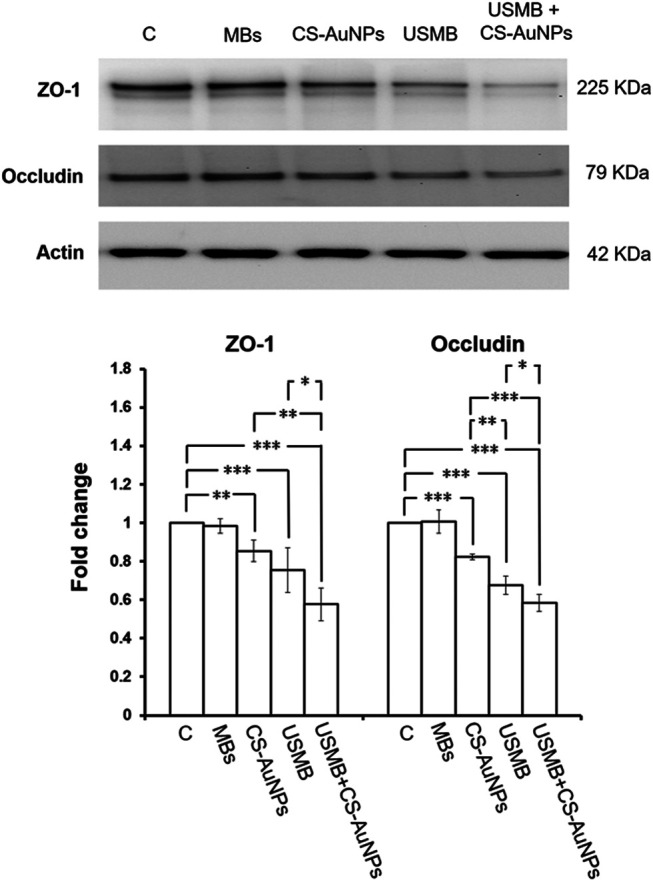
Representative western blot analysis of ZO-1 and occludin protein levels of the RWM **(upper panels)** after CS-AuNPs or USMB-aided CS-AuNPs treatments and digital quantification **(lower panels)**. The extracted proteins of untreated RWM served as control. The treatment period was 30 min for CS-AuNP soaking alone or for CS-AuNP soaking following a prior USMB exposure. Both ZO-1 and occludin protein amounts in the RWM were reduced after the CS-AuNPs treatment. This reduction was noticeable in the group given a USMB exposure alone and in the group subjected to CS-AuNPs soaking after a prior USMB exposure. The results are expressed as the mean ± standard error of the mean for the fold change in protein expression level relative to the control, with *n* = 4 for each bar. **p < 0.01; ***p < 0.001; C, control; MBs, microbubbles; Immed, immediate; control, mouse group not exposed to USMBs; US, ultrasound; USMB, ultrasound microbubble.

CS-AuNPs could also penetrate the RWM without prior USMB treatment although this penetration was significantly less efficient than that assisted by USMB (Figure 8). We were therefore interested in investigating whether CS-AuNPs treatment alone would disturb the TJ proteins of the RWM. As shown in Figure 13 **,** in the group treated with CS-AuNPs alone (30 min) showed significant reduction in the levels of ZO-1 and occludin compared to the untreated control but smaller reductions compared with the USMB treatment group. However, both TJ proteins were reduced further when CS-AuNPs treatment was combined with a prior USMB exposure. These data suggest that either CS-AuNPs or USMB alone could impair the TJ proteins, thereby affecting the paracellular permeability of the RWM. When AuNP treatment was assisted by USMB, this combination could have synergistic disruptive effects on TJ proteins and achieve greater permeability.

### Preservation of Hearing Thresholds After Chitosan-Coated Gold Nanoparticles, Ultrasound Microbubbles Treatment, or Combination Treatments

**FIGURE 14:**
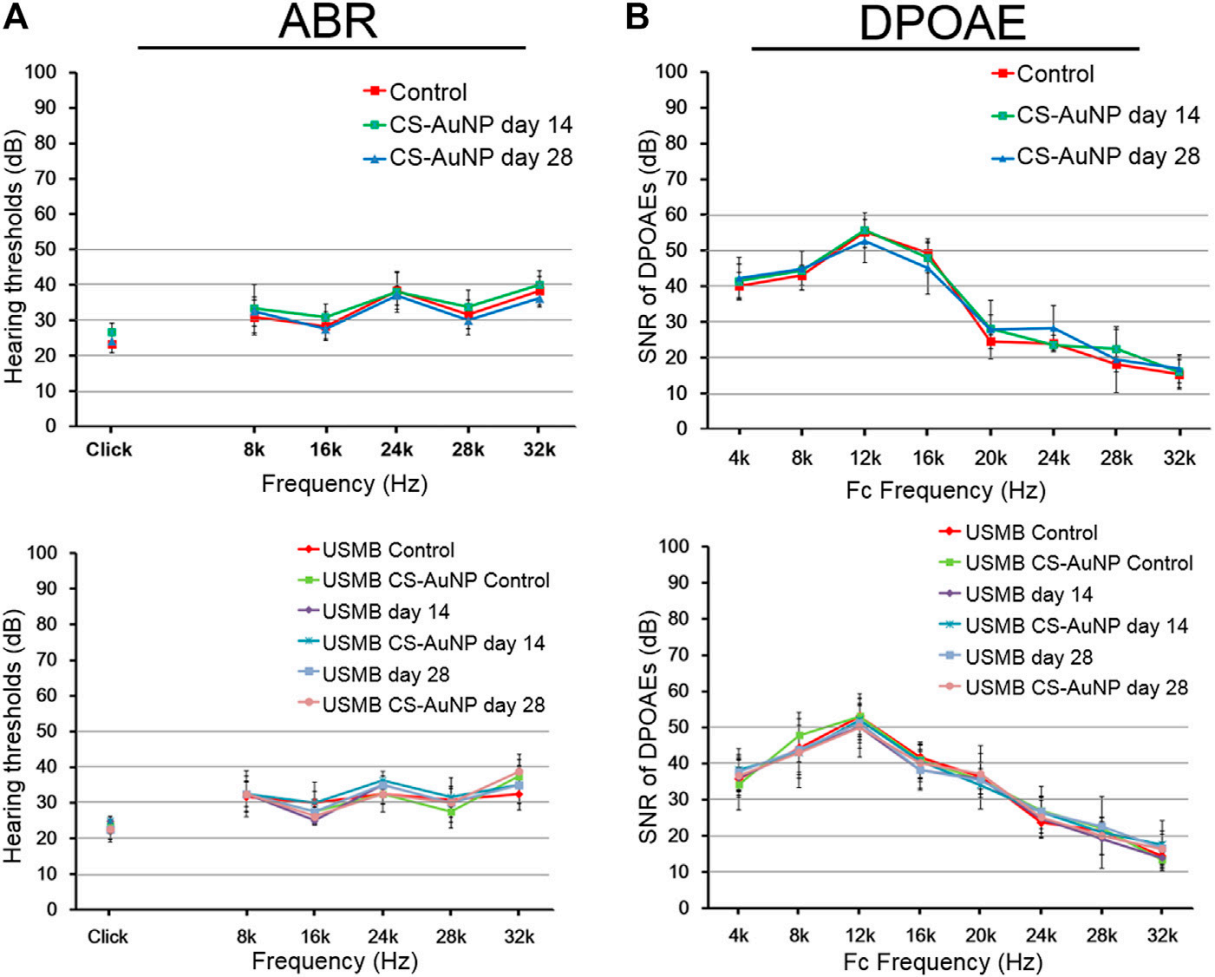
Hearing assessment in mice treated with CS-AuNPs with or without prior USMB exposure. **(A)** The ABR threshold recordings during the 28-day follow-up in the RWS and USM groups before (day 0) and after USMB exposures. **(B)** SNRs of the cubic difference distortion product (2F_1_–F_2_) at various center frequencies (F_C_) for each group. The results are expressed as the mean ± standard error of the mean, with *n* = 4 for each bar. DPOAE, distortion-product otoacoustic emission; USMB, ultrasound microbubble; CS-AuNP, chitosan-coated gold nanoparticle; ABR, auditory brainstem response; SNR, signal-to-noise ratio.

We investigated whether USMB intervention and CS-AuNP treatments might compromise the animals’ hearing thresholds by conducting ABR hearing assessments, which included both click-evoked and tone burst–evoked sounds at frequencies of 8, 16, 24, 28, and 32 kHz. During a 28-day follow-up, hearing did not differ between animals that received CS-AuNPs only, USMB only, or USMB combined with CS-AuNPs (Figure 14A). The SNR of the distortion-product measurements in each group at frequencies from 4 to 32 kHz exhibited no significant group differences during the 28-day follow-up (Figure 14B).

## Discussion

The mechanisms underlying the enhanced permeability of the RWM mediated by USMBs are not yet fully understood, and only a few studies have investigated this phenomenon (Lin et al., 2019; Zhang et al., 2021). USMB-induced cavitation is generally acknowledged to damage the RWM through extensive disruption of the outer epithelial cells; this damage occurred both following five courses of USMB sonication at an MI of 0.254 (Lin et al., 2019) and following continuous 5-min USMB sonication at 0.5 MI (Zhang et al., 2021). However, this damage may be limited to only the outer epithelium of the RWM. In this study, we investigated a reduced acoustic intensity of 2 W/cm^2^, estimated to be 0.207 MI, and applied it in the mouse model. The resultant ultrastructural changes in the outer epithelium were evidently less severe compared with previous US damage observed in a guinea pig model (Lin et al., 2019; Zhang et al., 2021). The physical explanation for this discrepancy lies in the fact that, at the same high frequency of 1 MHz, increases in the sound pressure at certain ranges achieve a higher cavitation activity or strength—the so-called cavitation intensity (Wu et al., 2020). For this reason, our results indicated that the cellular uptake of FITC-CS-AuNPs is proportional to the increase in the acoustic intensity in the range of 1–2 W/cm^2^; however, the increased intensity has no adverse effects on cell viability. By contrast, at 3 W/cm^2^, the sonication cavitation effect may produce extremely high shear forces that can break up the HEI-OC1 cells, negatively affect cell viability, and reduce the uptake of fluorescent FITC-CS-AuNPs. In terms of USMB-induced enhancement of cellular uptake, the mechanism has been demonstrated to occur through enhanced endocytosis, as evident from the observation that the uptake of FITC dextrans into a cell was colocalized with lysotracker (an endolysosomal marker) (Roovers et al., 2019).

NPs can enter the body through inhalation, skin, or digestive routes. Therefore, exploring the nanoscale interactions between NPs and the epithelia that serve as the portal of entry into the target organs or the body is essential from a practical perspective. NPs can use multiple pathways to enter and traverse cell monolayers, as revealed in an intestinal epithelial cell model (He et al., 2013b; Bannunah et al., 2014). Distinct molecular mechanisms, including endocytosis, exocytosis, and transcytosis processes, have also been carefully studied using polymer NPs in a canine kidney epithelial cell line model (He et al., 2013a). The consensus reached by these earlier studies is that NPs may display nonspecificity in terms of their endocytosis routes through the epithelial cells. Similarly, in the RWM model, NPs were internalized predominantly through macropinocytosis and caveolae-mediated endocytic pathways (Zhang et al., 2018). However, in the present study, we further demonstrated that AuNPs might also disrupt the TJ proteins of the RWM to enhance paracellular permeability.

Previous studies have shown that AuNP treatment could diminish the integrity of TJs and reduce the expression of TJ proteins, thereby increasing endothelial permeability (Li et al., 2015). One of the underlying mechanisms appears to involve a marked reduction in PKCζ-dependent threonine phosphorylation of occludin and ZO-1 by AuNP treatments, which caused an instability of endothelial TJs, followed by proteasome-mediated degradation of TJ components. Another study indicated that AuNPs possibly regulate the TJ barrier function of Caco-2 cell monolayers through a reversible redistribution of CLDN1 and ZO-1 proteins (Leve et al., 2019). Further experiments are needed to establish which models are most likely to be responsible for the AuNP-mediated TJ protein reduction in the RWM.

Molecules can cross tissues by traveling through cells (transcellular transport) or between cells (paracellular transport). The transcellular pathway, widely distributed on the larger accessible surface of the epithelium, provides the dominant entry route for most drugs (Pade and Stavchansky, 1997) and is thus a favored pathway for NPs to enter the RWM (Zhang et al., 2018). The paracellular space is sealed by TJs, which are maintained by a complex network of protein interactions (Buckley and Turner, 2018). Related studies have explored several strategies to increase the permeability of the RWM. Examples include using external magnetic forces to enhance transportation of superparamagnetic iron oxide NPs across an artificial RWM (Mondalek et al., 2006), manipulating drug solution osmolarity or benzyl alcohol content to alter the permeability properties of the RWM (Mikulec et al., 2008), and creating microperforations in the RWM to enhance diffusion across the membrane (Kelso et al., 2015; Chiang et al., 2020). All these attempts have focused on modifying the transcellular pathway of the RWM. In this study, we determined that USMB-mediated permeability enhancement involved the transcellular pathway, as evident from the pore-like openings and epithelial peeling observed in the ultrastructural analysis of the RWM. The paracellular pathway also plays a role, particularly via TJ-mediated paracellular transport.

TJs are a multifunctional complex that contribute to the paracellular barrier and limit intercellular fluxes of ions and molecules by forming a seal between adjacent epithelial cells near the apical surface (Farquhar and Palade, 1963). They are not static barriers but are highly dynamic structures that often undergo remodeling in response to interactions with external stimuli, such as temperature (Dokladny et al., 2006), extracellular calcium (Ma et al., 2000), and tumor necrosis factor-alpha (Ma et al., 2004). The claudin family of transmembrane proteins oligomerize into strands and interlock with their counterparts on neighboring cells to selectively regulate passage through the paracellular space (Varadarajan et al., 2019). The junctional adhesion molecules (JAMs), together with claudin strands, help limit macromolecule fluxes (Otani et al., 2019). The ZO proteins can multimerize with themselves and with other ZO proteins and subsequently bind claudins, JAMs, F-actin, and scaffolding proteins to facilitate the formation of a dense plaque of proteins associated with TJs (Beutel et al., 2019; Varadarajan et al., 2019).

A growing body of literature has examined the effect of US sonication on the TJ of the blood–brain barrier (BBB). The BBB is composed of specialized endothelial cells linked to each other by TJs. Following USMB sonication, the expression levels of ZO-1, occludin, and claudin-5 were decreased in parallel with disruption of the BBB (Zhao et al., 2018). Other work has also demonstrated a redistribution and loss of the immunosignals for occludin, claudin-5, and ZO-1 at the TJ of the brain microvascular endothelium following treatment of focused US applied with a contrast agent (Sheikov et al., 2008). In this study, we observed substantial reductions in both ZO-1 and occludin in the RWM after USMB exposure. In addition, increasing the number of USMB exposures to six or nine courses seemed to induce greater losses of both proteins compared with three exposures.

The underlying cause of the USMB-associated and exposure course–dependent loss of perijunctional proteins in the RWM merits further investigation; however, the accumulated mechanical damage to the TJ of the outer epithelium that resulted from USMB-mediated cavitation clearly contributed to this tissue damage and protein loss. This TJ disruption may depend on the cavitation intensity, as demonstrated by the ultrastructural and permeability analysis in this study and in our previous work (Lin et al., 2019). Notably, the lost proteins, ZO-1 and occludin, were almost completely recovered 3 days after treatment. Sheikov et al. noted a similar effect on the TJ of the BBB following focused US treatment, determining that the disassembled and lost junctional protein complexes were restored 24 h after sonication (Sheikov et al., 2008).

Evidence of the reassembly of TJ-associated protein complexes suggests that the TJ disruption due to the effect of either USMB or focused US may be a transient phenomenon. Studies investigating the activated leak pathway of the TJ in the US-exposed BBB have revealed that the duration of leakage ranged from 2 to 6 h, followed by complete closure after approximately 24 h (Sheikov et al., 2008; Jalali et al., 2010; Bae et al., 2015). By contrast, previous work in our laboratory indicated that USMB-mediated permeability changes in the RWM were greatest immediately after sonication and began to drop at 2 h, followed by a prolonged enhancement even after 72 h (Lin et al., 2019). This discrepancy may reflect the disparate US settings and power intensities applied to the BBB and the RWM and whether the transcellular pathway was involved in addition to paracellular TJ transport. Nevertheless, the return of TJ-associated proteins to pretreatment levels, as revealed in this study, confirms that the effects of USMBs on the RWM are transient.

The RWM is concave toward the tympanic cavity, convex toward the cochlea, and attached on the bony ridge of the round window. That cavitation effects on this uneven surface would differ in various locations is a matter of course. This is the first study to reveal that the middle and upper third areas of the RWM exhibit the strongest response to cavitation. Furthermore, the size of the affected area accounts for approximately one-quarter of the whole RWM, in agreement with a recent study conducted in guinea pigs that indicated that the damaged region was focused in the one-third area located anteriorly on the RWM (Zhang et al., 2021).

The RWM is a three-layered membrane. Unlike the intestines and most targeted organs, in which the epithelium is formed by a single layer of epithelial cells, NPs introduced into the RWM must penetrate two additional cellular layers to cross the whole membrane and reach the destination: the scala tympani of the cochlea. Conceivably, the transport process for NP-mediated drug and gene delivery through the RWM approach is more complex and diverse. However, the USMB treatment and cavitation effect creates a mechanical action that transiently modifies the permeability of the outer epithelial barrier and may lead to more efficient inner ear drug or gene delivery by NPs. We propose that the application of USMBs in combination with CS-AuNPs to the RWM would enable NPs to bypass the intracellular trafficking process of the outer epithelium cells, and these NPs can then exit from the basolateral membrane through exocytosis. Upon reaching the connective tissue layer, the NPs might further move directly through “gaps” among inner epithelial cells toward the final destination−the scala tympani (Zhang et al., 2018).

To the best of our knowledge, this is the first report to demonstrate that USMBs can facilitate the delivery of CS-AuNPs into the inner ear through a RWM approach. An improved understanding is needed of the CS-AuNP conjugates that can be used as carriers for genes that can be expressed in the inner ear following delivery via the USMB-created pathway through the RWM.

## Conclusion

The results presented here indicate that USMBs have an impressive efficiency in facilitating AuNP delivery to the inner ear. The mechanisms underlying the efficient delivery and the enhanced permeability of the RWM appear to involve transient disruption of the outer epithelium barrier resulting from the cavitation-induced opening of the transcellular pathway and the TJ-created paracellular barrier in the outer epithelium cells. Applying USMBs together with CS-AuNPs in the tympanic cavity did not damage the integrity of the RWM nor did it deteriorate the hearing thresholds. These promising features support the future clinical potential of this technique to provide a more efficient, feasible, and relatively noninvasive means of delivery for drug and gene therapeutics to the inner ear through the RWM.

## Data Availability

The original contributions presented in the study are included in the article/Supplementary Material, further inquiries can be directed to the corresponding author/s.
