# Supplementary material for: Ultrasound Microbubble–Facilitated Inner Ear Delivery of Gold Nanoparticles Involves Transient Disruption of the Tight Junction Barrier in the Round Window Membrane
Source: Front Pharmacol. 2021 Jun 28;12:689032. doi: 10.3389/fphar.2021.689032 (PMC8273281; doi:10.3389/fphar.2021.689032)
Supplement: Supplementary file 1 [file Image1.pdf]

Supplementary Figure S1:

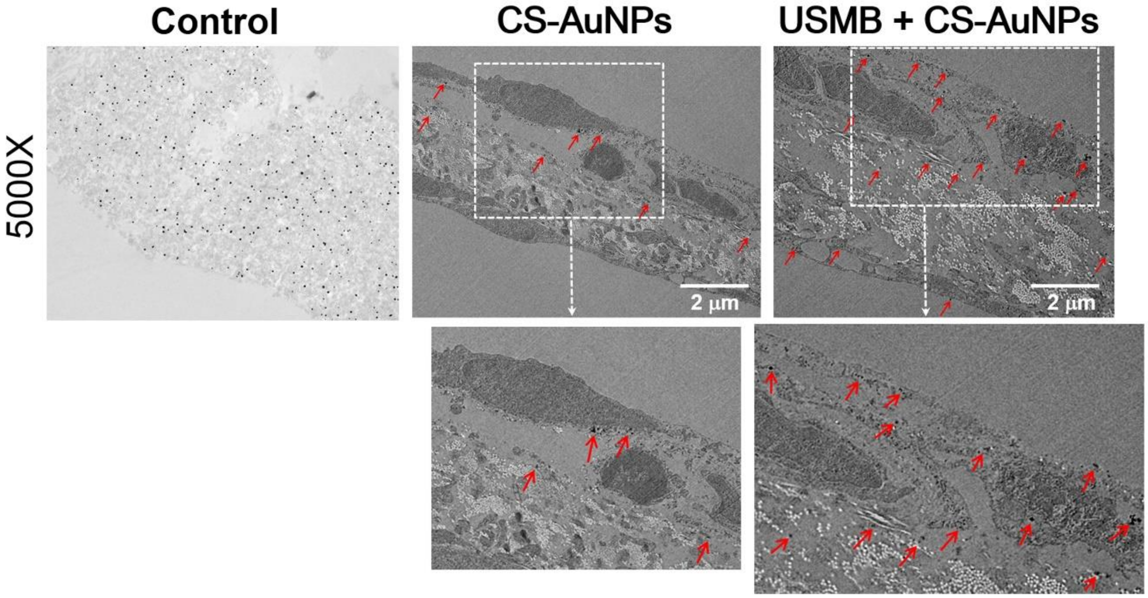

Supplementary Figure S1: Transmission electron micrographs of CS-AuNPs-treated round window membrane with prior USMB exposure. Cs-AuNPs-loaded gelatin sponge serves as a control. The dotted arrow-indicated photo shows a magnification of the square region, where gold nanoparticles can be visualized (red arrows).
